# Results of sclerotherapy and mucopexy with haemorrhoidal dearterialization in II and III degree haemorrhoids. A 4 years' single centre experience

**DOI:** 10.3389/fsurg.2023.1151327

**Published:** 2023-06-19

**Authors:** Pierluigi Lobascio, Rita Laforgia, Angela Pezzolla

**Affiliations:** Coloproctology Unit “Bari 2”, Laparoscopic and Emergency Surgical Unit, Department of Emergency and Trasnsplantation of Organs, Hospital University of Bari, Bari, Italy

**Keywords:** haemorrhoidal disease, mucopexy and dearterialization, sclerotherapy, goligher classification, office-based procedures, surgical treatment

## Abstract

**Introduction:**

Haemorrhoidal disease (HD) affects a considerable portion of the adult population. The aim of this study is to confirm the safety and efficacy of the treatments and to report the long-term outcomes of Sclerotherapy (ST) and Mucopexy and Haemorrhoidal Dearterialization (MHD) performed over the last 4 years in a single tertiary centre. The secondary outcome is to evaluate the usefulness of both techniques and to demonstrate how those can be associated as a bridge to surgery.

**Materials and methods:**

Patients affected by second–third-degree haemorrhoids and undergoing ST or non-Doppler guided MHD between 2018 and 2021 were enrolled. Safety and efficacy, recurrence rate, Haemorrhoid Severity Score (HSS) and pain resulting from both techniques were evaluated.

**Results:**

Out of 259 patients, 150 underwent ST. Further, 122 (81.3%) patients were male and 28 (18.7%) were female. The mean age was 50.8 (range 34–68) years. Most of the patients (103, 68.6%) were affected by second-degree HD, while 47 (31.4%) were affected by third-degree HD. The overall success rate was 83.3%. The median pre-operative HSS score was 3 (IQR 0–4, *p* = 0.04) and at 2 year the median HSS was 0 (IQR 0–1, *p* = 0.03). No intraoperative complications and no drug-related side effects occurred. The mean follow-up for ST was 2 years (range 1–4; SD ±0.88). MHD was performed on 109 patients. In detail, 80 patients (73.4%) were male while 29 patients (26.6%) were female. The mean age in this group was 51.3 (range 31–69). Further, 72 patients (66.1%) were affected by third-degree HD and 37 (33.9%) by second-degree HD. The median HSS score was 9 (IQR 8–10, *p* = 0.001) preoperatively two years after treatment was 0 (IQR 0–1, *p* = 0.004). Major complications occurred in three patients (2.75%). The overall success rate was 93.5% (second degree 89.2% vs. third degree 95.8%). The mean follow-up for MHD was 2 years (range 1–4; SD ±0.68).

**Conclusions:**

The results confirm the usefulness of those techniques, which can be considered safe and easily repeatable procedures, with a low recurrence rate after 2 years of median follow-up.

## 1. Introduction

Haemorrhoidal disease (HD) is one of the most common proctological diseases affecting the general population, from mid-teens onward, with considerable implications for the National Health Service (NHS) in terms of cost and surgeons' workload (1).

Despite various classifications having been proposed over the last 50 years, currently, Goligher's classification is still the most used, driving the diagnosis and representing the best therapeutic option for each patient (2). In the last two decades, several new techniques and devices have been proposed for HD treatments (3).

According to European guidelines (2), sclerotherapy (ST) can be recommended for first-, second- and third-degree HD when conservative treatment fails. A local intravenous injection of sclerosant agents induces sclerosis of the submucosal tissue with endothelial damage of the vessels and consequent suspension of the haemorrhoidal tissue (4).

Different sclerotherapy techniques with various sclerosant agents have been described in the literature. Nowadays, polidocanol is the most frequently used sclerosant agent because it is a non-ionic surfactant that targets endothelial cells (5).

The advantage of ST is the possibility to perform the procedure in an outpatient setting and to repeat the treatment “on demand”. Furthermore, this technique could be adopted as a bridge to surgery, especially in high-risk patients.

An improved understanding of the pathogenesis of haemorrhoids and of the complications associated with excisional haemorrhoidectomy has led to the invention of new surgical procedures, including mucopexy with haemorrhoidal dearterialization (MHD), with or without Doppler, which can be used for second-, third- and, in certain cases, even for fourth-degree HD. This technique consists of interrupting the vascular supply and lifting the haemorrhoidal cushions. This technique has shown encouraging results in terms of postoperative pain, complications and long-term recurrence (6).

The aim of this study is to confirm the safety and efficacy of the treatments and to report the long-term outcomes of ST and MHD performed over the last 4 years in a single tertiary centre. The secondary outcome is to evaluate the usefulness of both techniques with different trends and results, and to demonstrate how those can be associated as a bridge to surgery.

## 2. Materials and methods

### 2.1. Patients

A retrospective study was designed to evaluate the safety, efficacy and long-term outcomes of sclerotherapy and mucopexy with non-Doppler guided haemorrhoidal dearterialization for symptomatic haemorrhoidal disease. The study was carried out in the Coloproctology Unit “Bari 2”, Hospital University of Bari, between February 2018 and December 2021 (including the first wave of the Sars-Cov-2 pandemic, when there was a decrease in surgical activities (7, 8). All patients were not enrolled consecutively and all procedures were performed by the same experienced surgeon.

The inclusion criteria were: second- to third-degree symptomatic haemorrhoids, including previous HD with evidence of recurrence. For ST, the inclusion criteria also included patients on a waiting list for surgery, HD associated with severe anaemia requiring blood transfusion (as an emergency procedure), as well as HD in patients refusing surgery with American Society of Anesthesiologists scores of 3 and 4.

Pregnant women, patients younger than 18 years old, those affected by external haemorrhoidal thromboses or by other proctological diseases or IBD patients were excluded.

Information regarding family history, bowel habits, diet and previous intake of flavonoids was collected before the proctological evaluation, consisting of digitorectal examination and anoscopy. The guidelines on perioperative management of anticoagulant and antiplatelet agents were applied according to pharmaceutical anamnesis.

The severity of the condition was assessed through the administration of the Haemorrhoid Severity Score (HSS) (9) and the Visual Analog Scale (VAS from 0 to 10) for pain assessment.

All patients were asked to fill in a daily diary for the first 7 postoperative days and to report bleeding, pain, soiling, tenesmus, return to daily activity and satisfaction grade. Follow-up was scheduled at 1, 3, 6, 12, 24 and 48 months in the outpatient clinic.

Written informed consent was obtained from all patients.

### 2.2. Surgical techniques

• *Sclerotherapy*

Sclerosant foam (Atossisclerol 3% alias Polidocanol-Lauromacrogol 400 by Gloria Med Pharma) was administered according to a modified Blonde-Blanchard technique. Polidocanol foam is injected directly into the haemorrhoids at the 3, 7 and 11 o'clock positions and not into the submucosa, above the dentate line, with 2.5 ml of foam injected into each pile. The inclination of the needle in male patients should be tangential to the 11 o'clock position pile to avoid prostatic tissue. The foam was obtained as previously described (10).

**Figure 1:**
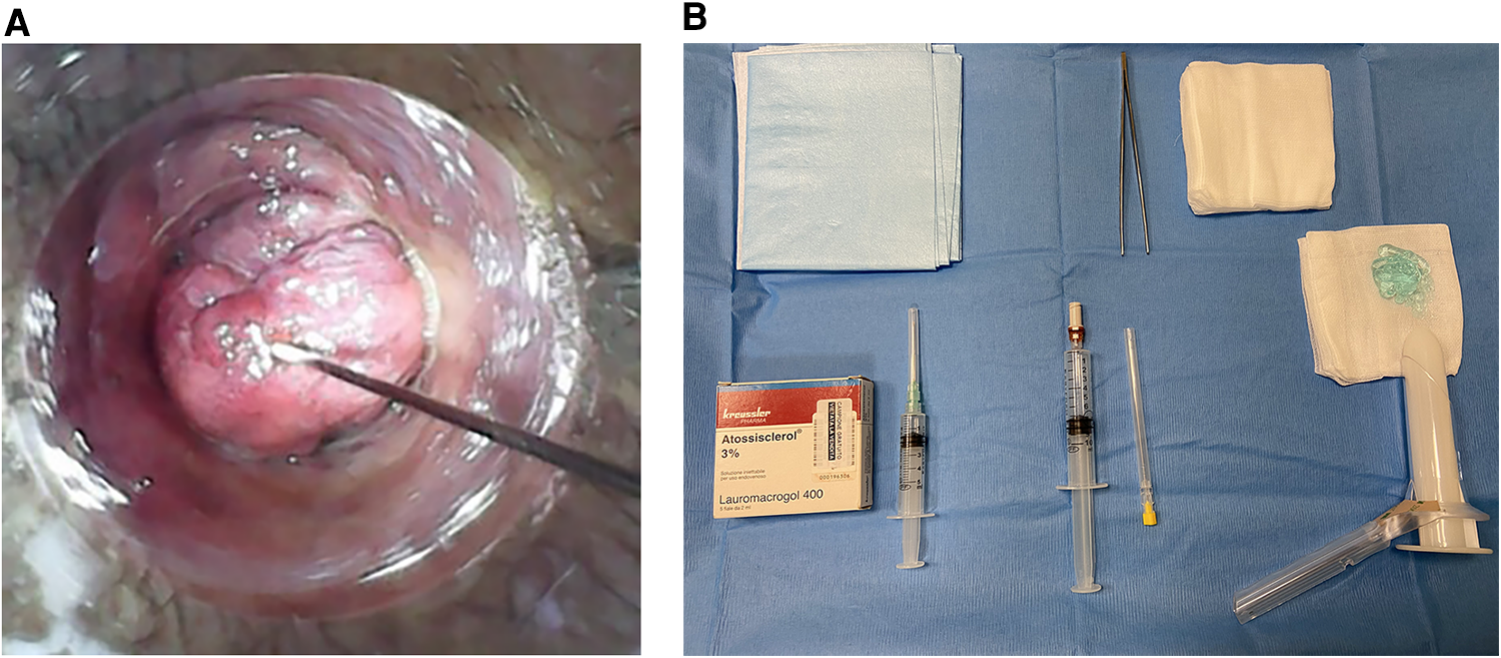
(**A,B**): sclerotherapy injection and materials.

The patients were treated in the Sims position with no anaesthesia. Walking for 20 min after the procedure was suggested before a pre-discharge check (Figures 1A,B).

• *Mucopexy and Haemorrhoidal Dearterialization with EndoRectal Operative Device*

**Figure 2:**
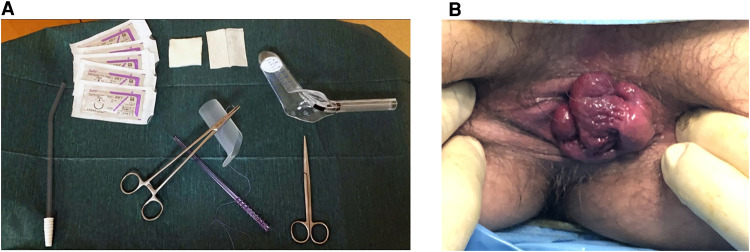
(**A,B**): mucopexy with haemorrhoidal dearterialization materials and pre-operative 3rd degree haemorrhoidal disease.

Mucopexy with haemorrhoidal dearterialization was performed without Doppler, using the EndoRectal Operative Device (ERODe—Copyright © Sapi Med S.P.A.)*.* Six longitudinal mucosal plications with 2–0 gauge, 5/8 Circle round-body needle, polygliactin sutures at 1, 3, 5, 7, 9 and 11 o'clock, not exceeding the dentate line, were performed, ligating arteries without a Doppler guide, with the patient in a modified lithotomy position and under spinal anaesthesia. In some cases, another plication was necessary because of excess mucosal prolapse or haemorrhoidal tissue. The number of single plication steps was variable from sector to sector in proportion to the mucosal prolapse. The ERODe device consists of a conic retractor with an oval distal part, with a plate that allows for variation in the depth of the socket. This device allows for the homogenous and progressive dilation of the anal canal, with optimum ergonomics and dexterity (Figures 2A,B).

Discharge was scheduled on the first post-operative day (POD), with careful dietary and defaecation recommendations.

### 2.3. Statistical analysis

All the data were collected in an Office Excel® sheet. The chi-square test was used to compare categorical variables. Odds ratios (ORs) and 95% CI were calculated when required. The Mann–Whitney *U* test was used to compare continuous variables not normally distributed (presented as median, interquartile range (IQR) and range). Normality of variable distribution was determined using the D'Agostino–Pearson test. A *p* value < 0.05 was considered to be statistically significant. All tests were two-sided.

Data were analysed using R Studio (Version 1.1.419—© 2009–2018 RStudio, Inc).

## 3. Results

In total, 259 patients were enrolled in this study: 150 were recruited in the ST group and 109 patients in the MHD group.

### 3.1. Sclerotherapy

First, 150 symptomatic patients with second- and third-degree haemorrhoids underwent sclerotherapy treatment.

Further, 122 (81.3%) patients were male, 28 (18.7%) were female and their mean age was 50.8 (range 34–68) years. Most of the patients (103, 68.6%) were affected by second degree HD, while 47 (31.4%) were affected by third-degree HD. No intraoperative complications and no drug-related side effects occurred.

**Figure 3:**
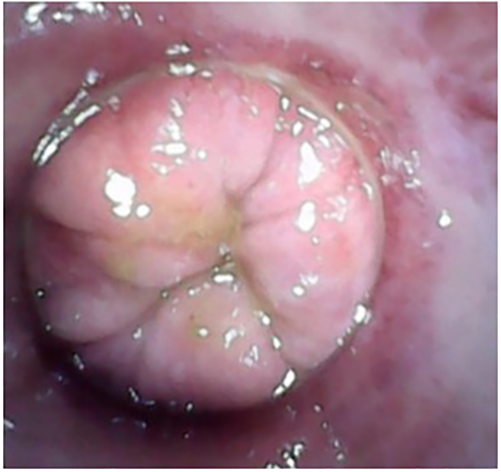
Follow-up after sclerotherapy (one month).

All patients resumed their normal daily activities the day after the procedure. The overall success rate was 83.3% after a single ST session (second degree 87.5% vs. third degree 73.9%) (Figure 3).

Recurrences in terms of bleeding occurred in 21 (31.5%) patients. A second ST session was performed for 11 patients (16.5%), and 10 patients required surgical treatment (Mucopexy and Dearterialization).

**Figure 4:**
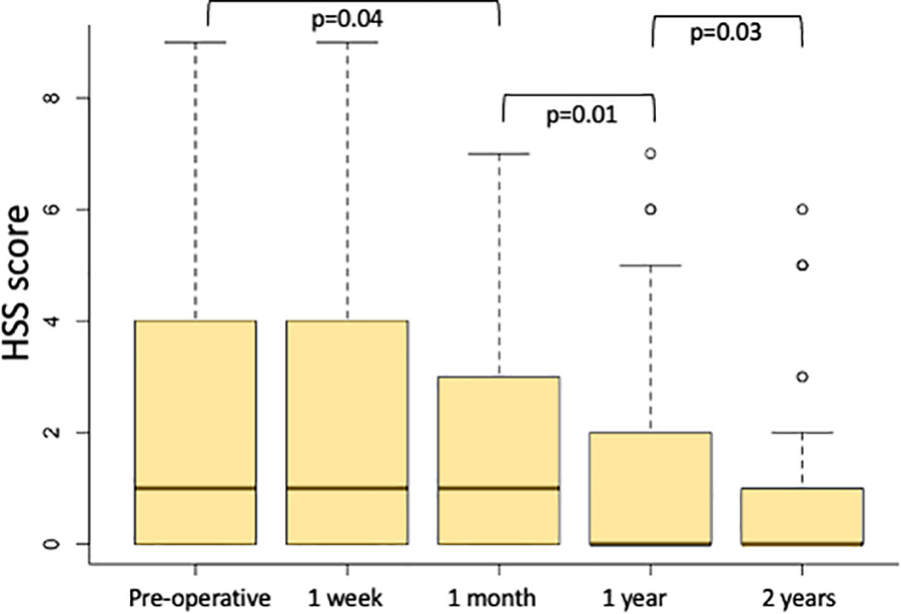
Haemorrhoids severity score (HSS) calculated preoperatively, one week, one month, one year and two years after ST.

The median pre-operative HSS score was 3 (IQR 0–4, *p* = 0.04) and it did not improve after one week (median 3, IQR 0–4, *p* = 0.04), while it significantly improved after one month (median 2, IQR 0–3, *p* = 0.01) and at the one-year follow-up, with a median of 1 (IQR 0–1, *p* = 0.01). The effectiveness of ST was also confirmed after a follow-up of 2 years, with a median HSS of 0 (IQR 0–1, *p* = 0.03) (Figure 4). Eleven patients (16.5%) were affected by severe anaemia (in one case haemoglobin was less than 7gr/dl) and required blood transfusions, and, in these cases, ST was performed in an emergency setting.

VAS score pain was 0 in all patients after the procedure and during all follow-up sessions. The mean operative time was 3 (2–5; SD ± 1.03).

The Hospital University of Bari was a COVID-19 centre from March 2020, and proctological activity was stopped for one year. Only 15 ST treatments were performed for symptomatic HD disease (second and third degree): two cases were affected by severe anaemia, requiring a blood transfusion.

The mean follow-up for ST was 2 years (range 1–4; DS ±0.88). Follow-up was regularly scheduled in an outpatient clinic with complete proctological examination.

All results can be appreciated in Table 1.

**Table 1:** Patient's characteristics.

	Sclerotherapy	MHD
**Patients**	150	109
M	122 (81.3%)	80 (73.4%)
F	28 (18.7%)	29 (26.6%)
Age	50.8	51.3
**Haemorrhoidal Disease Degree**		
Second	103 (68.6%)	37 (33.9%)
Third	47 (31.4%)	72 (66.1%)
**Overall Success Rate**		
Second	87.5%	89.2%
Third	73.9%	95.8%
**Recurrences**	21 (31.5%)	7 (4.6%)
**Haemorrhoid Severity Score (HSS)**		
pre op	2.4	9
1 week	2.3	6
1 month	1.4	5
1 year	1	2
2 years	0	0
**Mean Hospital stay (days)**	0	1.1
**Mean Operative Time (min)**	3	40

### 3.2. Mucopexy with haemorrhoidal dearterialization

In terms of patients, 109 underwent this surgical procedure. Further, 80 patients (73.4%) were male while 29 patients (26.6%) were female. The mean age in this group was 51.3 (range 31–69). Additionally, 72 patients (66.1%) were affected by third-degree HD and 37 (33.9%) by second-degree HD.

Fourteen patients (12.8%) had refractory HD, treated by previous surgical or outpatient procedures; eleven patients (10.1%) were also affected by anterior rectocele; and six patients (5.5%) had severe anaemia.

The mean hospital stay was 1.1 days (IQR 1–5), and the mean operative time was 40 min (IQR 34–52).

**Figure 5:**
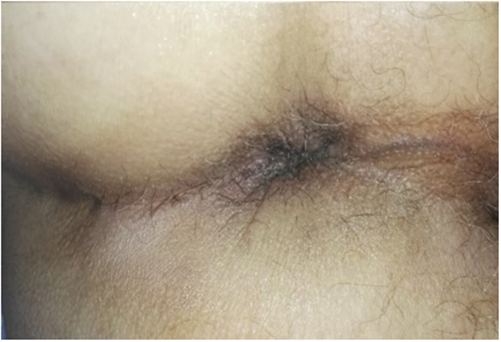
Follow-up after modified mucopexy with haemorrhoidal dearterialization (one year).

The median VAS pain score was 5 (IQR 3–8) on the 7th postoperative day (POD) and less than 2 after the first 10 days. In the first week after the procedure, bleeding occurred in 14 patients (15.2%), while tenesmus was reported by 66 patients (60.5%). At one month follow-up, bleeding was reported by three patients (2.75%) and tenesmus by two patients (2.1%) (Figure 5).

**Figure 6:**
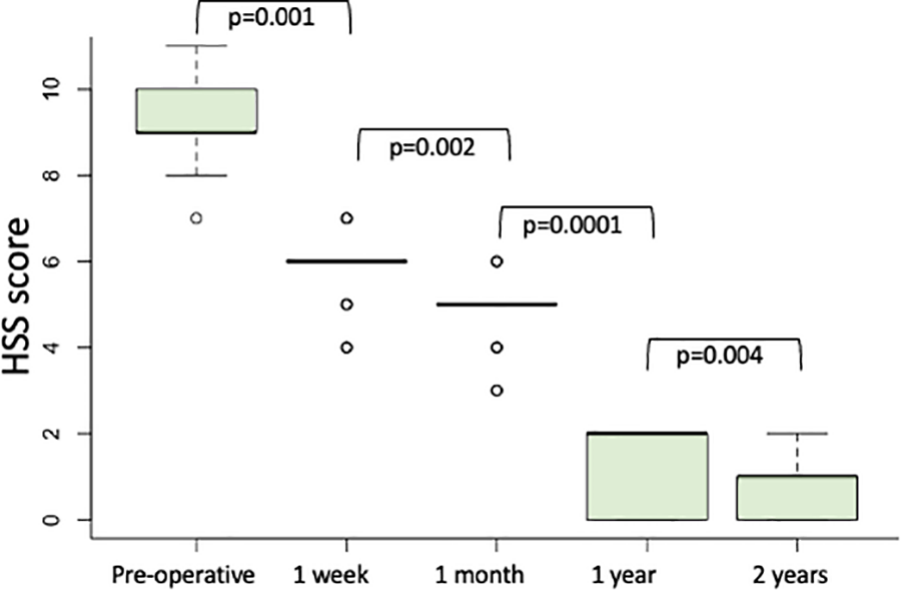
Haemorrhoids severity score (HSS) calculated preoperatively, one week, one month, one year and two years after modified mucopexy with haemorrhoidal dearterialization.

The median HSS score was 9 (IQR 8–10, *p* = 0.001) preoperatively and it significantly improved after one week (median value 6, IQR 5–6, *p* = 0.002), progressively decreased at one month (median value 5, IQR 3–6, *p* = 0.001) and after one year (median value 2, IQR 0–2, *p* = 0.0001). The HSS score also improved two years after treatment (median value 0, IQR 0–1, *p* = 0.004) (Figure 6).

Major complications, consisting mainly of haemorrhage, occurred in three cases (2.7%), and these were treated with a blood transfusion in two cases and *via* surgical revision in one patient. Minor complications, including haemorrhoidal thrombosis, occurred in six (6.6%) patients, while abscess formation was observed in one patient. In this patient, the abscess was surgically drained in an outpatient setting. Recurrences were noted after 6 months in seven patients (4.6%). Three patients with a recurrent prolapse were treated *via* re-do surgery. Four patients reported persistent bleeding and they were treated with ST.

The overall success rate was 93.5% (second degree 89.2% vs. third degree 95.8%). The mean follow-up for MHD was 2 years (range 1–4; SD ±0.68). Follow-up was regularly scheduled in the outpatient clinic with a complete proctological examination.

All results can be appreciated in Table 1.

## 4. Discussion

The management of haemorrhoids has significantly changed in the last few decades. New insights into their pathophysiology have been described, and new mini-invasive surgical devices and procedures have been proposed (11).

Some of these procedures have been validated and included in national and international guidelines (2, 12).

A first step in the treatment of HD should include dietary changes (adequate water intake, high-fibre diet, laxatives such as bulking agents) and flavonoid intake. Patients with symptomatic HD should be informed of the pros and cons of all procedures, and patient shared decision making is crucial (2, 12).

Among the outpatient treatments, there are a few options that can be proposed to patients (13).

James Morgan described the use of Sclerotherapy for the first time in history of HD in 1869 (14).

The composition of the foam injected during ST has been a matter of debate. In 2000, Petrin reported good results with polidocanol 1% in 80 patients affected by second-degree HD (15).

The comparison between the transanal approach vs. endoscopic ST is still debated in the literature (16). Nevertheless, recent studies suggest better outcomes and comfort for patients treated with transanal ST rather than endoscopic ST (17).

In this study, we used the transanal approach, injecting Atossisclerol 3%, which has previously been demonstrated to be a safe, effective and repeatable conservative treatment for HD (4, 5, 18–21).

Furthermore, the modified Blonde-Blanchard technique adopted in this report reduces the risks of major complications, such as compartment syndrome, necrotising fasciitis, retroperitoneal sepsis, rectourethral fistula (22–25) and prostate injury, that can lead to prostatitis or prostatic abscess (26). The adopted technique with a tangential approach to the 11 o'clock pile is beneficial in that it avoids the prostatic tissue.

The results showed that after ST, pain is well-controlled, while tenesmus seems to be a frequent symptom until 7 POD (60.5%), which may be due to the oedema and hypertension in each of the haemorrhoidal piles. Furthermore, the reported patients demonstrated significant improvements in terms of bleeding in second-degree HD and also in terms of prolapse in third-degree HD.

Although Keng-Sheong reported “poor” long-term outcomes in terms of recurrence rate after ST (27), in this study, we reported a low recurrence rate after a period of at least 2 years.

The second group of patients was treated with a modified technique involving Mucopexy with Haemorrhoidal Dearterialization using the ERODe device without a Doppler guide. In the last decade, some studies have demonstrated the efficacy of haemorrhoidal artery ligation if associated with rectoanal repair or mucopexy for third-degree haemorrhoids (28, 29). On the other hand, Aigner et al. (30) recently cast some doubt on the usefulness of Doppler-guided haemorrhoidal artery ligation and reported that the Doppler proof was not beneficial for third-degree haemorrhoid treatment when compared to suture mucopexy alone.

This study demonstrates that the “suspensive” technique using the ERODe device without a Doppler guide is safe and repeatable for both prolapse and bleeding. In fact, plication lifts the haemorrhoidal and mucosal tissue, and artery ligation reduces bleeding. According to the Italian Society of Colorectal Surgery consensus statement, the use of MHD with or without Doppler is associated with a faster recovery, less postoperative pain and good outcomes in eligible patients when compared to excisional procedures (12). A Doppler guide could be helpful to identify the haemorrhoidal arteries, but it is not mandatory and does not modify outcomes (1, 2, 12).

A recent multicentre study reported benefits from Doppler-guided MHD using a THD device with a 9.5% recurrence and 7% reoperation rate (29, 31, 32). Giuliani et al. reported that MHD is a safe and efficient technique, especially for third-degree HD (33).

The results of this work exposed a lower recurrence rate and shorter surgery duration in a smaller sample of recruited patients. Furthermore, MHD failure can be treated using ST in terms of bleeding resolution.

ST and MHD are two different procedures, not comparable in terms of application and surgical technique, but they may be associated. Eligibility criteria for each procedure can be combined in cases of recurrences in second-degree and third-degree HD and as a bridge to surgery or re-do surgery.

The statistical analysis demonstrates that there are no differences in terms of significance, reporting a good overall success rate for both procedures over the last 4 years.

This study has some limitations: it is a retrospective, single-centre study, based on the description of results of two treatments for HD, without any comparison to similar procedures, and the follow-up is not homogeneous. Patients were not selected consecutively representing a selection bias. This study presents real-world evidence from an experienced proctologic centre, accomplished according to the Strengthening the Reporting of Observational Studies in Epidemiology (STROBE) checklist (34).

## 5. Conclusions

The results of this study from an experienced proctologic centre confirm the usefulness of Sclerotherapy with Atossisclerol 3% and Mucopexy with Haemorrhoidal Dearterialization using ERODe device and demonstrate how they can be combined. Those techniques can be considered safe and easily repeatable procedures, with a low recurrence rate after 2 years of median follow-up.

## Data Availability

The raw data supporting the conclusions of this article will be made available by the authors, without undue reservation.

## References

[1] Picciariello A Tsarkov PV Papagni V Efetov S Markaryan DR Tulina I Classifications and clinical assessment of haemorrhoids: the Proctologist's Corner. Rev Recent Clin Trials. (2021) 16(1):10–6. 10.2174/1574887115666200312163940 32164517

[2] van Tol RR Kleijnen J Watson AJM Jongen J Altomare DF Qvist N European Society of ColoProctology: guideline for haemorrhoidal disease. Colorectal Dis. (2020) 22(6):650–62. 10.1111/codi.14975 32067353

[3] Altomare DF Picciariello A Pecorella G Milito G Naldini G Amato A Surgical management of haemorrhoids: an Italian survey of over 32 000 patients over 17 years. Colorectal Dis. (2018) 20(12):1117–24. 10.1111/codi.14339 30004171

[4] Lobascio P Laforgia R Novelli E Perrone F Di Salvo M Pezzolla A Short-Term results of sclerotherapy with 3% polidocanol foam for symptomatic second- and third-degree hemorrhoidal disease. J Invest Surg. (2021) 34(10):1059–65. 10.1080/08941939.2020.1745964 32290709

[5] Gallo G Pietroletti R Novelli E Sturiale A Tutino R Lobascio P A multicentre, open-label, single-arm phase II trial of the efficacy and safety of sclerotherapy using 3% polidocanol foam to treat second-degree haemorrhoids (SCLEROFOAM). Tech Coloproctol. 26(8):627–36. 10.1007/s10151-022-02609-w 35334004 PMC8949823

[6] Ratto C Campennì P Papeo F Donisi L Litta F Parello A. Transanal haemorrhoidal dearterialization (THD) for hemorrhoidal disease: a single-center study on 1000 consecutive cases and a review of the literature. Tech Coloprocol. (2017) 21(12):953–62. 10.1007/s10151-017-1726-5 PMC5830492 29170839

[7] Gallo G Sturiale A De Simone V Mancini S Di Tanna GL Milito G Deadlock of proctologic practice in Italy during COVID-19 pandemic: a national report from ProctoLock2020. Updates Surg. (2020) 72(4):1255–61. 10.1007/s13304-020-00860-0 32770466 PMC7414270

[8] Bracale U Podda M Castiglioni S Peltrini R Sartori A Arezzo A Changes in surgicaL behaviOurs dUring the COVID-19 pandemic. The SICE CLOUD19 study. Updates Surg. (2021) 73(2):731–44. 10.1007/s13304-021-01010-w 33656697 PMC7926077

[9] Nyström P-O Qvist N Raahave D Lindsey I Mortensen N. Randomized clinical trial of symptom control after stapled anopexy, or diathermy excision for haemorrhoid prolapse. Br J Surg. (2010) 97(2):167–76. 10.1002/bjs.6804 20035531

[10] Lobascio P Minafra M Laforgia R Giove C Trompetto M Gallo G. The use of sclerotherapy with polidocanol foam in the treatment of second-degree haemorrhoidal disease—a video vignette. Colorectal Dis. (2019) 21(2):244–5. 10.1111/codi.14498 30485654

[11] Gallo G Ronconi M Trompetto M. Sclerotherapy with 3% polidocanol foam: revolutionizing outpatient treatment in patients with haemorrhoidal disease. Updates Surg. (2021) 73(5):2029–30. 10.1007/s13304-021-01008-4 33660166 PMC7927757

[12] Gallo G Martellucci J Sturiale A Clerico G Milito G Marino F Consensus statement of the Italian society of colorectal surgery (SICCR): management and treatment of hemorrhoidal disease. Tech Coloproctol. (2020) 24(2):145–64. 10.1007/s10151-020-02149-1 31993837 PMC7005095

[13] Tomasicchio G Martines G Lantone G Dibra R Trigiante G De Fazio M Safety and effectiveness of tailored hemorrhoidectomy in outpatients setting. Front Surg. (2021) 8:708051. 10.3389/fsurg.2021.708051 34485375 PMC8415450

[14] Pata F Gallo G Pellino G Vigorita V Podda M Di Saverio S Evolution of surgical management of hemorrhoidal disease: an historical overview. Front Surg. (2021) 8:727059. 10.3389/fsurg.2021.727059 34527700 PMC8435716

[15] Petrin P Segalina P Canton A Rupolo G Farinea L. Significance of patients’ satisfaction with an ambulatory treatment: experience with sclerotherapy of haemorrhoids. Int J Surg. (2000) 1:2–10.

[16] Ronconi M Casiraghi S Schieppati M. EndoTHeF: endoluminal treatment of hemorrhoids with foam. Ann Colorectal Res. (2019) 6(4):e86297. 10.5812/acr.86297

[17] Skowronski A Diaco E Trompetto M Gallo G. Use of video-gided sclerotherapy with 3% polidocanol foam for symptomatic second-degree haemorrhoidal disease—a video vignette. Colorectal Dis. (2020) 22(9):1198–9. 10.1111/codi.15040 32180308

[18] Moser K-H Mosch C Walgenbach M Bussen DG Kirsch J Joos AK Efficacy and safety of sclerotherapy with polidocanol foam in comparison with fluid sclerosant in the treatment of first-grade haemorrhoidal disease: a randomized, controlled, single-blind, multicentre trial. Int J Colorectal Dis. (2013) 28(10):1439–47. 10.1007/s00384-013-1729-2 23775099

[19] Gallo G Picciariello A Pietroletti R Novelli E Sturiale A Tutino R Sclerotherapy with 3% polidocanol foam to treat second-degree haemorrhoidal disease: three-year follow-up of a multicentre, single arm, IDEAL phase 2b trial. Colorectal Dis. (2023) 25(3):386–95. 10.1111/codi.16380 36268758

[20] Gallo G Pietroletti R Novelli E Sturiale A Tutino R Lobascio P A multicentre, open-label, single-arm phase II trial of the efficacy and safety of sclerotherapy using 3% polidocanol foam to treat second-degree haemorrhoids (SCLEROFOAM). Tech Coloproctol. (2022) 26(8):627–36. 10.1007/s10151-022-02609-w 35334004 PMC8949823

[21] Goglia M Nigro C Aurello P Diaco E Trompetto M Gallo G. Preliminary results of the first 50 patients undergoing sclerotherapy for II-degree hemorrhoidal disease using an automated device. Front Surg. (2022) 9:882030. 10.3389/fsurg.2022.882030 35495738 PMC9046905

[22] Yang P Wang YJ Li F Sun JB. Haemorrhoid sclerotherapy with the complication of abdominal compartment syndrome: report of a case. Chin Med J. (2011) 124(12):1919–20. 21740855

[23] Schulte T Fandrich F Kahlke V. Life-threatening rectal necrosis after injection sclerotherapy for haemorrhoids. Int J Colorectal Dis. (2023) 25(3):386–95. 10.1007/s00384-007-0402-z 18043926

[24] Barwell J Watkins RM Lloyd-Davies E Wilikins DC. Life threatening retroperitoneal sepsis after haemorrhoid injection sclerotherapy: report of a case. Dis Colon Rectum. (1999) 42(3):421–3. 10.1007/BF02236364 10223767

[25] Gupta N Katoch A Lal P Hadke NS. Rectourethral fistula after injection sclerotherapy for haemorrhoids, a rare complication. Colorectal Dis. (2011) 13(1):105. 10.1111/j.1463-1318.2009.02156.x 20002694

[26] Namasivayam J Payne D Maguire D. Prostatic abscess following injection of internal haemorrhoids. Clin Radiol. (2000) 55(1):67–8. 10.1053/crad.1999.0066 10650114

[27] Kheng-Seong N Holzgang M Young C. Still a case of “No pain, No gain"? an updated and critical review of the pathogenesis, diagnosis, and management options for hemorrhoids in 2020. Ann Coloproctol. (2020) 36(3):133–47. 10.3393/ac.2020.05.04 32674545 PMC7392573

[28] Giordano P Overton J Madeddu F Zaman S Gravante G. Transanal haemorrhoidal dearterialization: a systematic review. Dis Colon Rectum. (2009) 52:1665–71. 10.1007/DCR.0b013e3181af50f4 19690499

[29] Ratto C Parello A Veronese E Cudazzo E D'Agostino E Pagano C Doppler-guided transanal haemorrhoidal dearterialization for haemorrhoids: results from a multicentre trial. Colorectal Dis. (2015) 17:910–9. 10.1111/codi.12779 25213152

[30] Aigner F Bodner G Conrad F Mbaka G Kreczy A Fritsch H. The superior rectal artery and its branching pattern with regard to its clinical influence on ligation techniques for internal hemorrhoids. Am J Surg. (2004) 187:102–8. 10.1016/j.amjsurg.2002.11.003 14706597

[31] Gachabayov M Angelos G Bergamaschi R. THD Doppler: a reliable surgical procedure to treat hemorrhoids. Surg-Technol Int. (2019) 34:189–93. 30888670

[32] Parello A Litta F De Simone V Campennì P Orefice R Marra AA Haemorrhoidal haemodynamic changes in patients with haemorrhoids treated using Doppler-guided dearterialization. BJS Open. (2021) 5(2):zrab012. 10.1093/bjsopen/zrab012 33839752 PMC8038259

[33] Giuliani A Romano L Necozione S Cofini V Di Donato G Schietroma M Excisional hEMOrrhoidectomy versus DeARTerialization with mucopexy for the treatment of grade 3 haemorrhoidal disease: the EMODART3 multicenter study. Dis Colon Rectum. (2023). 10.1097/DCR.0000000000002885 (Forthcoming). 37616177

[34] von Elm E Altman DG Egger M Pocock SJ Gøtzsche PC Vandenbroucke JP STROBE Initiative.1111/codi.12779. The strengthening the reporting of observational studies in epidemiology (STROBE) statement: guidelines for reporting observational studies. Lancet. (2007) 370(9596):1453–7. 10.1016/S0140-6736(07)61602-X 18064739

